# Exploring the Interactive Effect of Pectins from *Campomanesia xanthocarpa* with Galectin‑3

**DOI:** 10.1021/acsomega.6c01637

**Published:** 2026-04-02

**Authors:** Isabela P. Dias, Gabriela Casani Cardoso, Lucas V. dos Santos, Giovanna Furman, Ester Mazepa, Téo F. Minella, Marcelo D. Baruffi, Luisa Mestriner, Andrey F. Z. Nascimento, Lindomar J. C. Albuquerque, Keylla L. Mischiatti, Edneia A. S. Ramos, Elaine C. de Abreu, Sheila M. Brochado Winnischofer, Sarah da Costa Amaral, Joana Léa Meira da Silveira, Guilherme F. Picheth

**Affiliations:** † Postgraduate Program in Biochemistry Sciences, 28122 Federal University of Paraná, Curitiba, Paraná 80060-000, Brazil; ‡ Department of Biochemistry and Molecular Biology, Federal University of Paraná, Curitiba, Paraná 80060-000, Brazil; § Department of Clinical, Toxicological and Bromatological Analysis, 28133 University of São Paulo, Ribeirão Preto, São Paulo 14040-903, Brazil; ∥ Brazilian Synchrotron Light Laboratory, Brazilian Center for Research in Energy and Materials, Campinas, São Paulo 13083-100, Brazil; ⊥ Post-Graduate Program in Microbiology, Parasitology and Pathology, Federal University of Paraná, Curitiba, Paraná 80060-000, Brazil; # Department of Basic Pathology, Federal University of Paraná, Curitiba, Paraná 80060-000, Brazil; ∇ Center for Advanced Fluorescence Technologies from the Federal University of Paraná - CTAF/UFPR, Curitiba, Paraná 81531-980, Brazil

## Abstract

Glioblastoma (GBM) is a fast-growing and aggressive primary brain tumor with an average survival time of 12–18 months. GBM growth and invasion rely on multiple and dynamic signaling events in the tumor microenvironment, in which several glycan-binding proteins act as essential mediators. Among them, galectin-3 (Gal-3) plays a key role in cell–cell and cell–extracellular matrix communication. Gal-3 may be inhibited by complex polysaccharides such as pectins, which might reduce GBM aggressiveness and invasiveness. To identify possible interactive mechanisms involving Gal-3, we have selectively modified the structure of pectins extracted from*Campomanesia xanthocarpa* Berg to obtain varied GalA:Ara and Gal:Ara ratios along the polysaccharide backbone. The interactive influence of the fully modified structures as well as the individual monosaccharides was monitored by circular dichroism and X-ray diffraction. The impact of all modified pectins on the T98G and U87MG cell viability and Gal-3 expression was also evaluated. Our results suggest that galactose and galacturonic acid may be partly responsible for the protein structural changes. The pectins induced significant cytotoxicity and reduced Gal-3 expression *in vitro*, suggesting that biomaterials with a similar Gal:Ara ratio may be employed as adjuvants in GBM therapy.

## Graphical abstract

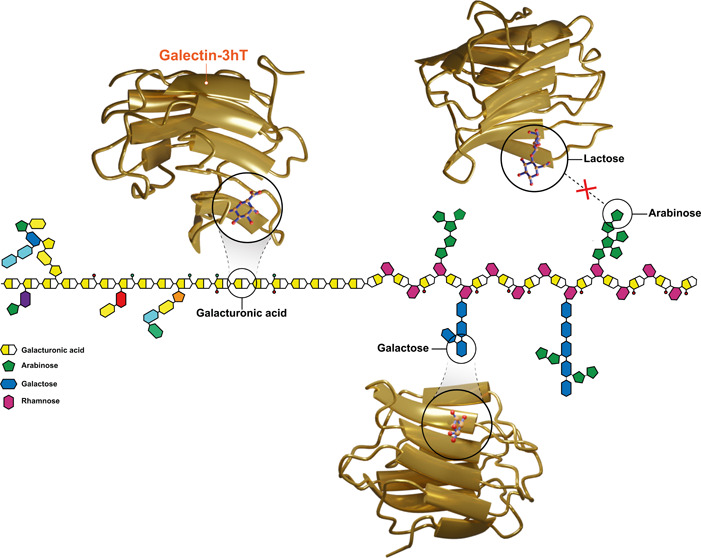

## 1 Introduction

Glioblastoma (GBM) is one of the most common and deadliest types of brain tumors.^1^ GBM is characterized by aggressive and malignant behavior (WHO grade IV) with an inherent refractory character toward chemoradiation and immunotherapy.^2,3^ The epidemiological data on GBM are worrying—6 cases per 100,000 individuals per year—with an average lifespan between 6 and 18 months after diagnosis and therapy.^4^ Such tumors are among the most feared types of cancer, not only due to their poor prognosis, but also because of direct repercussions on the patient’s life quality and cognitive function.^5^

GBM development and spread are intimately related to the local microenvironment—abundantly comprised by microglia and infiltrated macrophages^6^—that acquire an anti-inflammatory phenotype that supports GBM growth.^7^ The proliferative signaling provided by the local tumor microenvironment is complex and dynamic; however, transcriptional as well as epigenetic regulation is intimately associated with altered glycosylation patterns^8^ and aberrant expression of several glycan-binding proteins that act as essential mediators for tumor growth and survival.^9^ This particular class of proteins, denominated as lectins, shares a common carbohydrate recognition domain (CRD) capable of establishing specific interaction with glycosylated components within the extracellular matrix (ECM).^10^ The human lectins are classified according to their subcellular location and structure, comprising the C-type, I-type, P-type, pentaxins, and S-type, also known as galectins.^11^

The galectin family is known to regulate several immune events by co-opting selected inhibitory receptors, disrupting costimulatory pathways, and/or controlling activation, differentiation, and survival of immune cells.^12^ Among them, several studies have identified higher expression rates and elucidated specific roles of galectin-3 (Gal-3), a β-galactoside-binding protein that was recently associated with a higher risk of GBM development as well as poor prognosis.^6,13^ Gal-3 is a multifunctional protein found in several cell compartments as well as the ECM, where it mediates adhesion and activation processes.^14,15^ In particular, Gal-3 is involved in several physiological and pathological phenomena by recognizing specific carbohydrates and transducing intra as well as extracellular signals.^16^

Structurally, Gal-3 consists of an intrinsically disordered N-terminal domain (NTD) that is linked to the CRD, which displays high specificity for β-galactosides.^17,18^ Nonetheless, unlike the other members of the galectin family, the biological activity of Gal-3 depends on an oligomerization event induced by the NTD that is triggered by carbohydrate binding at the CRD.^19^ Recent reports indicate that Gal-3 expression is modulated by hypoxia and nutrient deprivation as an adaptive strategy for tumor development in the central nervous system.^20^ Accordingly, Gal-3 signaling induces an immunosuppressive state that, ultimately, favors GBM migration and invasion.^21^ The emergence of new evidence highlighting the central role of Gal-3 in driving GBM development and progression compels the search of new therapeutic agents especially designed for Gal-3 inhibitors.

Pectins were previously identified as high-affinity Gal-3 inhibitors^22^ of particular interest for their low toxicity and wide availability.^23^ The pectin structure is mainly composed of (1 → 4)-linked-α-d-GalA*p* units, consisting of homogalacturonan (HG) and rhamnogalacturonan type-I (RG-I), with a backbone of repeating [→2)-α-L-Rha*p*-(1 → 4)-α-d-GalA*p*-(1→]_n_ disaccharide units, which may contain neutral monosaccharide side chains predominantly composed of galactose and arabinose.^24,25^ Nonetheless, the interactive events that modulate the binding of α(1 → 4)-linked polymers with a β-galactoside-binding protein are not well understood.

To further investigate the structural features enrolled on pectin:Gal-3 inhibition, we performed specific chemical modifications on pectins extracted from *Campomanesia xanthocarpa* Berg—previously identified as a GBM inhibitory pectin^26^—for obtaining varied GalA:Ara and Gal:Ara ratios. The interactive potential of structural elements as well as the pectins in solution with Gal-3 was analyzed by X-ray diffraction and circular dichroism. In addition, the impact of the polysaccharide structure on U87MG and T98G cell viability and Gal-3 expression was assessed *in vitro*.

## 2 Materials and Methods

### 2.1 Materials

Ripe *C. xanthocarpa* Berg fruits were collected in Irati, Paraná, Brazil—geographic coordinates of 25° 25′ south latitude, 50° 36′ west longitude, and 25° 17′ south latitude, 50° 30′ west longitude.^27,28^ The fruits were selected and washed, the peel and seeds were removed, and the pulp was stored at −20 °C. The commercial pectin Genu type LM-104 AS was purchased from CP Kelco (Brazil). Absolute grade ethanol (P.A. ≥ 99.9%) was purchased from VETEC (Duque de Caxias, Brazil). HCl 1 mol·L^–1^ (1 N) was purchased from Neon (Suzano, Brazil). NaCl (≥99%) and CaCl_2_ (≥99%) were purchased from Êxodo Científica (Sumaré, Brazil). Inositol (myo-Inositol, ≥99%), *m*-hydroxydiphenyl (phenyl phenol, ≥90%), NaBH_4_ (≥98.0%), NaOAc (≥99%), NaOH (≥97%), NaNO_2_ (≥99%), pyridine (≥99%), the standards glucose (Glc—purity ≥ 99%), galacturonic acid (GalA—purity ≥ 97%), glucuronic acid (GlcA—purity ≥ 99%), arabinose (Ara—purity ≥ 99%), rhamnose (Rha—purity ≥ 99%), xylose (Xyl—purity ≥ 99%), mannose (Man—purity ≥ 99%), and trifluoroacetic acid (TFA, ≥99%) were purchased from Sigma-Aldrich (St. Louis, USA). The ultrapure water (18 MΩ·cm, pH 6.9) was obtained through the Master system-MS2000/Gehaka.

### 2.2 Pectin Extraction and Purification

The pectin extraction and purification process was performed as previously reported by Barbieri et al.^27^ and da Costa Amaral et al.,^29^ respectively. In brief, the pulp (∼1.5 kg) was thawed, dispersed in 99% ethanol, and submitted to reflux for 30 min in a water bath at 60 °C. The solid residue containing the polysaccharides was then filtered to remove the solvent and resuspended in ultrapure water. Afterward, the mixture was exposed to a boiling water bath under reflux for 4 h, cloth filtered and centrifuged at 12,000 × *g*, 4 °C for 25 min. The supernatant was collected, concentrated via rotary evaporation, and precipitated by adding 99% ethanol (3:1, v/v). The precipitated polysaccharides were dialyzed (6–8 kDa cellulose membranes, Spectrum Laboratories) against ultrapure water for 48 h, followed by three washing steps with ethanol, filtered, and dried under vacuum for 24 h—this fraction was denoted as GW. The resulting material was purified by dissolving 1 g of the dry polysaccharides in 100 mL of ultrapure water overnight and centrifuging it at 4,600 × *g*, 20 °C for 1 h to remove insoluble particles. The supernatant was collected and 0.45 g of NaCl (0.08 mol·L^–1^) was added to the solution that was stirred for 30 min to obtain the sodium salt of the polysaccharides. Afterward, the pH was adjusted to 7.0 using HCl (0.1 mol·L^–1^) and the solution was centrifuged at 4,600 × *g*, 20 °C during 1 h. The supernatant was collected, precipitated with 99% ethanol (50% vol) and subsequently washed by an ethanol gradient in four steps (from 50% to 99%) in a cloth filter for complete water removal. Finally, the precipitated material was dried in a vacuum desiccator for 24 h—this fraction was denoted as HM.

### 2.3 Pectin De-esterification

The HM sample was further submitted to a de-esterification process by dissolving 150 mg of the previously purified material in 24 mL of ultrapure water overnight at 4 °C. Afterward, 6 mL of NaOH 0.1 mol·L^–1^ was added and the solution was homogenized for 30 min in room temperature. Following, the pH was adjusted to 7 using HCl 1 mol·L^–1^ and the product was precipitated in ethanol and dried as previously described—this fraction was denoted as LM.

### 2.4 Pectin Characterization

The elution profiles of the samples were determined by high-performance size-exclusion chromatography (HPSEC) coupled with multiangle static light scattering (MALS; Wyatt Technology, Santa Barbara, CA, USA), dynamic light scattering (DLS; WyattQELS module, Wyatt Technology, Santa Barbara, CA, USA), and a refractive index detector (RI; Waters 2414, Milford, MA, USA). The chromatographic analyses were performed using a Waters system equipped with four Ultrahydrogel columns (2000, 500, 250, and 120) connected in series, with exclusion limits of 7 × 10^6^, 4 × 10^5^, 8 × 10^4^, and 5 × 10^3^ g mol^–1^, respectively. The mobile phase consisted of 0.1 mol·L^–1^ NaNO_3_ containing 0.5 g·L^–1^ NaN_3_, delivered at a flow rate of 0.6 mL.min^–1^ at 25 °C. Prior to injection, the samples were filtered through 0.22 μm membranes and injected (100 μL) at a concentration of 1 mg·mL^–1^. Data acquisition and analysis were carried out using ASTRA software (version 8.2.2.115, Wyatt Technology).

Monodimensional (^1^H, ^13^C) and bidimensional (HSQC) NMR spectra were acquired at 70 °C with a Bruker Avance III 400 MHz or Bruker Avance III HD 600 MHz spectrometers, equipped with a BBI 5 mm probe (400 MHz) or with a 5 mm CPP-TCI probe (600 MHz) (Bruker, USA). The samples were dissolved in D_2_O and the chemical shifts of the polysaccharide were expressed as δ (ppm), using the resonances of −CH_3_ groups of acetone (^1^H at δ 2.22; ^13^C at δ 30.20) as internal references. The data were collected and processed using the Software TOPSPIN, version 3.1 (Bruker Biospin, Germany).

The hydrodynamic diameter of pectin was determined by dynamic light scattering (DLS) at 632.8 nm using a Zetasizer Nano Series ZS and PANalytical (Egham, Surrey, United Kingdom). Measurements were performed at a sample concentration of 100 μg·mL^–1^ in 0.1 mol·L^–1^ sodium nitrate buffer containing 300 ppm sodium azide. Data acquisition was performed using Zetasizer Nano v. 3.30 software.

#### 2.4.1 Monosaccharide Composition

The assessment of neutral monosaccharides was conducted via total acid hydrolysis using 2 mol·L^–1^ TFA for 8 h at 100 °C. The resulting hydrolyzates were then transformed into alditol acetates by treatment with NaBH_4_ ^30^ followed by acetylation using acetic anhydride (Ac_2_O)-pyridine (in a 1:1, v/v ratio, 1 mL) at 100 °C for 30 min. The alditol acetates obtained were extracted with CHCl_3_ and subjected to analysis using a Thermo Scientific Trace GC Ultra gas chromatograph. The chromatograph employed a carrier gas mixture of He, N_2_, and compressed air at a flow rate of 1 mL·min^–1^. The DB-225-MS column (0.32 mm internal diameter × 30 m × film thickness 0.25 μm) was used and the system was programmed to increase the temperature from 100 to 230 °C at a heating rate of 60 °C·min^–1^. The identification of the alditol acetates was based on their profile, comparing retention times with established standards. The quantification of uronic acid content within the pectin fraction was conducted employing the colorimetric *m*-hydroxybiphenyl method, utilizing galacturonic acid as the standard reference with concentration of 10–100 μg·mL^–1^ analyzed at 520 nm on an SP-22 BioSpectro spectrophotometer.^31^

#### 2.4.2 Methyl-Esterification Quantification

The degree of methyl-esterification was quantified using a conductometric titration technique as previously described,^29^ with modifications. In brief, 20 mg of the sample was dissolved in 50 mL of deionized water and solubilized overnight. Then, to determine the uronic acid content in free carboxylic acid, titration was performed with 0.02 mol·L^–1^ HCl solution followed by titration with a 0.02 mol·L^–1^ NaOH, using a conductometer (MS Tecnopon Instrumentação, mCA 150). The degree of methyl-esterification (DM) was calculated by the ratio between GalA-Met and total GalA expressed in molarity × 100, as usually adopted (eq 1). Additionally, from those determinations, the fraction of neutral carbohydrates (corresponding to the neutral side chains of the pectins in the sample) can be deduced by the difference between the total mass of the sample and the total GalA content. And finally, to convert the percentages obtained from g/g to mol/g, the values were normalized considering the molar mass of 198 g·mol^–1^, 190 g·mol^–1^, and 162 g·mol^–1^ for GalA^–^ Na^+^, GalA-Met, and neutral monosaccharides, respectively.

1 $$DM=\frac{GalAMet\;(mol)}{Total\;GalA\;(mol)}\times 100$$

The degree of methyl-esterification was also determined by ^1^H NMR spectroscopy by integrating the hydrogen areas corresponding to H-1, H-5 of unesterified α-d-Gal units and H-5 of esterified α-d-Gal units.^32^ The hydrogen from −CH_3_ around 3.76 ppm gives DM using the H-1 integral as reference.

### 2.5 Expression and Purification of Recombinant Human Galectin-3

Recombinant human galectin-3 (Gal-3h) and recombinant human truncated galectin-3 (Gal-3hT) were produced in *E. coli* BL21/DE3 transformed with the pET11a plasmid with the human galectin-3 (hrGal-3) coding DNA or the C-terminal domain fragment of galectin-3 (hrGal-3T).^33^ The *E. coli* cells were grown in Luria–Bertani (LB) medium supplemented with 100 μg·mL^–1^ of ampicillin in an inoculum overnight at 37 °C, 180 rpm. Then, this suspension was transferred into 1 L of LB medium supplemented with 100 μg·mL^–1^ of ampicillin and cultivated at 37 °C and 180 rpm until the optical density (OD at 600 nm) reached 0.45. Protein expression was achieved by the addition of 500 μM isopropyl β-d-1-thiogalactopyranoside (IPTG) followed by culturing for 4 h at 37 °C, 180 rpm. Finally, the suspension was centrifuged at 10,000 × *g* for 10 min at 4 °C. The obtained cell pellets were lysed (Lysis buffer: PBS, lysozyme, DNase, RNase, and protease inhibitor cocktail) for 1 h at 4 °C, sonicated, and then centrifuged at 10,000 × *g* for 10 min at 4 °C. The soluble portion (supernatant) was collected and loaded into a lactosyl-Sepharose resin (Sigma) followed by a wash with phosphate-buffered saline (PBS) supplemented with 14 mM of 2-mercaptoethanol (PBS-2-ME). The proteins were eluted with PBS-2-ME supplemented by 100 mM lactose and quantified in a NanoDrop 2000 device using the NanoDrop 2000 software, version 1.4.1 (Thermo Fisher Scientific, USA). Lactose and 2-ME were removed from protein preparations using a PD-10 gel-filtration column, and the solutions were kept at 4 °C until use. All samples were sterilized by 0.22 μm polyvinylidene fluoride (PVDF) filtration.

The recombinant proteins were characterized by SDS-PAGE, using a 12% SDS-polyacrylamide gel under reducing conditions.^34^ The molecular weight was estimated by a protein marker. The gel was stained with Coomassie Blue R-250. Quantitative analysis was made by Bradford method.^35^ The samples were also analyzed by the hemagglutination assay by exposing the proteins to erythrocytes as previously described.^36^

#### 2.5.1 Crystallization of Gal-3hT

The Gal-3hT was concentrated to 8.8 mg·mL^–1^ in the purification buffer (PBS with 14 mM 2-mercaptoethanol). Initial screening for crystallization conditions was performed based on the previous crystallization solutions for Gal-3hT reported in the Protein Data Bank (PDB) and commercial kits. The sitting-drop vapor-diffusion plates were prepared by the Honey Bee 963 system (Genomic Solutions) by mixing 0.5 μL of the crystallization condition and 0.5 μL of the protein solution at 8.0 or 8.8 mg·mL^–1^ and kept at 18 °C until the crystals grew (3–5 days). The best crystals were obtained with a protein concentration of 8.0 mg·mL^–1^ with the following conditions: 0.1 M Tris-HCl pH 7.5, 33% w/v PEG 6000, 0.1 M magnesium chloride, 0.008 M 2-mercaptoethanol (native and galactose soak) and 0.1 M Tris-HCl pH 7.0, 31% w/v PEG 6000, 0.1 M Magnesium chloride, 0.008 M 2-mercaptoethanol (galacturonic acid soak). Before the diffraction data collection, the crystals were quick-soaked in a cryoprotective solution of 25% v/v PEG 400 and flash-frozen into liquid nitrogen.

#### 2.5.2 Gal-3hT X-ray Diffraction and Data Analysis

The X-ray diffraction data were collected at 100 K on the MANACÁ beamline at the Sirius synchrotron (LNLS, CNPEM, Campinas) using a PILATUS 2M detector (Dectris Ltd.) and an X-ray wavelength of 0.97718 Å. The diffraction images were processed using XDS^37^ (and the data were cut based on CC_1/2_).^38^ The Gal-3hT crystal structure was solved by molecular replacement with PHASER^39^ using the PDB ID 3ZSJ as the search model. The models were built and refined using COOT^40^ and phenix refine.^41^ The final models were validated based on the agreement with diffraction data and stereochemical parameters using Molprobity.^42^ The three models obtained in this study, Gal-3hT native (lactose), galactose, and galacturonic acid soak, were deposited in the PDB under the codes 9D62, 9D63, and 9D64, respectively.

#### 2.5.3 Circular Dichroism

The circular dichroism (CD) experiments were performed at the Cedro Beamline (Brazilian Synchrotron Light Laboratory, LNLS, Campinas, Brazil) using a nitrogen-flushed OLIS DSM20 UV/vis spectropolarimeter (OLIS, USA). First, Gal-3hT or Gal-3h was dissolved at 0.05 mg·mL^–1^ in PBS buffer (pH 7.4) and measured alone. Then, Gal-3hT or Gal-3h with different monosaccharides (arabinose—Ara, galactose—Gal, and galacturonic acid—GalA), the disaccharide lactose (β-d-galactopyranosyl-(1→4)-d-glucose), and Gabiroba pectins (GW, LM, and HM) were added at a concentration of 150 μg·mL^–1^. The samples were studied across the far-UV region of 190–250 nm. Measurements were taken with an integration time of 1 s, using a 0.2 mm path length quartz cell and a 1 nm bandwidth at 37 °C. Five replicates were acquired for each sample, and baseline corrections were made by subtracting the buffer contribution. The resulting data were processed using CDToolX and BeStSel.^43^

### 2.6 *In Vitro* Cytotoxicity

T98G and U87MG human glioblastoma cell lines were kindly provided by the Mammalian Cells Collection of the Federal University of Paraná (INCT-CERBC). The cells were cultured in DMEM high-glucose medium, supplemented with 10% fetal bovine serum (Gibco, Thermo Fisher Scientific, USA), and maintained at 37 °C in a 5% CO_2_ atmosphere. The cells were seeded in 96-well culture plates at a density of 1 × 10^4^ cells per well. Following a 24 h adhesion period, the cells were treated with varying concentrations (50, 100, 200, 300, 400, 500, 700, and 1000 μg·mL^–1^) of the investigated pectins (GW, HM, and LM) and the commercial sample diluted in growth medium for 48 h. The MTT (3-(4,5-dimethylthiazol-2-yl)-2,5-diphenyltetrazolium bromide) (Invitrogen, Thermo Fisher Scientific, USA) assay was conducted after the treatment by replacing the medium with a 0.5 mg·mL^–1^ MTT solution. The plates were then shielded from light and incubated at 37 °C in a 5% CO_2_ atmosphere for 3 h. Afterward, the MTT medium was removed, and the formazan crystals were dissolved in dimethyl sulfoxide (DMSO). The absorbance was measured at 545 nm using a microplate reader spectrophotometer (Kasuaki, Japan). The results were expressed as a percentage of the control, designated as 100% of metabolically active cells. The subcytotoxic dose was selected as the optimal concentration for subsequent assays, aiming to identify cellular alterations preceding cell death.

### 2.7 Confocal Laser Scanning Microscopy

For microscopy analysis samples, T98G and U87MG cells were cultivated onto autoclaved glass slides for 24 h and incubated with the subcytotoxic dose (500 μg·mL^–1^ for T98G; 300 μg·mL^–1^ for U87MG) of GW, HM, LM, and the commercial sample during 48 h. Afterward, the cells were washed with PBS, fixed with paraformaldehyde 2% (m/v) for 20 min at 4 °C and washed with PBS. The blocking was made with BSA 5% (Invitrogen, Thermo Fisher Scientific, USA) with 0.01% of saponin overnight. The nucleus was stained with 4′-6-diamidino-2-phenylindole (DAPI) (Invitrogen, Thermo Fisher Scientific, USA) and the cytoskeleton was labeled with anti-Actin Red 555 (Invitrogen, Thermo Fisher Scientific, USA). The GAL-3 protein was labeled with the anti-GAL-3 primary antibody (Invitrogen, Thermo Fisher Scientific, USA; 1:100 dilution) in BSA/Saponin solution overnight, followed by the incubation with the secondary antibody Anti-Rat IgG 488 Alexa Fluor (Invitrogen, Thermo Fisher Scientific, USA; 1:2000 dilution) in BSA/Saponin solution for 1 h. The slides were examined by laser scanning confocal multiphoton microscope, model A1MP + (Nikon, Tokyo, Japan), using a 60× objective (numerical aperture 1.40, oil immersion). Fluorescence imaging was acquired and stacks were collected every 0.5 μm along the *z*-axis. All images were captured with the software Nis Elements 4.20 (Nikon, Tokyo, Japan) and measurements were processed using ImageJ software v1.51n.

### 2.8 Western Blot Assay

Cells were seeded in 100 cm^2^ culture plates at a density of 1 × 10^6^ cells per plate. After a 24 h adhesion period, the cells were treated for 48 h with the subcytotoxic dose of GW, HM, LM, and the commercial sample (500 μg·mL^–1^ for T98G and 300 μg·mL^–1^ for U87MG). Following treatment, cells were washed with ice-cold PBS 1X and total proteins were extracted with lysis buffer supplemented with MS-SAFE protease and phosphatase inhibitors (Sigma-Aldrich, Merck, Germany). Protein concentration was determined using the Bradford assay (Bio-Rad, USA). Ten micrograms of total protein were resolved on a 12% SDS–PAGE gel and transferred onto a PVDF membrane (Amersham, GE Healthcare). Membranes were blocked with 5% BSA in TBS-T buffer (Tris 2 M, NaCl 0.15 M, Tween-20 0.1%) and incubated with primary antibodies, anti-GAL-3 (Invitrogen, Thermo Fisher Scientific, USA; 1:800 dilution) or anti-β-actin (Invitrogen, Thermo Fisher Scientific, USA; 1:1000 dilution) in 5% BSA/TBS-T. Subsequently, membranes were incubated with HRP-conjugated secondary antibodies, antirat (Santa Cruz Biotechnology, USA; 1:5000 dilution) or antimouse (Invitrogen, Thermo Fisher Scientific, USA; 1:2000). The β-actin protein was used as a loading control for normalization. Chemiluminescence detection was performed using SuperSignal West Femto substrate (Thermo Fisher Scientific, USA) and images were analyzed with Lab Image software (Bio-Rad, USA). For the analysis, the signal intensity of each band was quantified based on the integrated optical density (IOD) generated by the software after background subtraction. For normalization, the intensity values of the target protein were divided by the intensity values of the corresponding endogenous control in the same lane. The normalized values were then used for comparative analysis between control and treated groups.

### 2.9 Statistical Analysis

The statistical analysis was conducted using one-way ANOVA and Tukey’s multiple comparison test. A *p*-value less than 0.01 was considered statistically significant. All data were presented as mean ± SD from at least three independent experiments. Statistical analyses were performed using GraphPad Prism 5.0 software (GraphPad Software Inc., USA).

## 3 Results and Discussion

### 3.1 Purification and Structural Characterization of Pectins and Galectin-3

The crude fraction of the gabiroba pectin (GW, ∼4% yield) was obtained according to the description of Barbieri et al.^44^ and subsequently purified (HM, 57% yield) as previously reported by da Costa Amaral et al.^26^ Following de-esterification, an LM pectin (89% yield) with distinct structural characteristics and a reduced degree of methyl-esterification was obtained.

The monosaccharide composition revealed that the GW structure is mainly composed by arabinose (Ara, 56 ± 0.2%), galacturonic acid (GalA, 28 ± 2.8%), and galactose (Gal, 11 ± 0.2%) (Table 1). Furthermore, all samples presented a greater proportion of RG-I segments (65 to 74%) compared to the HG region (23 to 32%), demonstrating that they are mostly branched. After the purification and de-esterification process, the monosaccharide composition analysis revealed that arabinose remains as the main unit for HM (Ara, 59 ± 0.06%), while its content is significantly reduced at the LM fraction (Ara, 49 ± 0.2%). The reduction in Ara content may be linked to the hydrolysis of pectin side chains, which are susceptible to the degradation process in alkaline environments.^45^ Conversely, the GalA amount is preserved after the purification and de-esterification processes, indicating that LM displays an increased GalA:Ara ratio compared to HM.

**Table 1:** Monosaccharide Composition (%) of Pectins from *C. xanthocarpa* Berg Pulp^a^

	Monosaccharide composition (%)								
Sample	GalA^b^	Rha	Ara	Xyl	Gal	Glc	Man	HG^c^	RG-I^c^
**GW**	28.5 ± 2.8	0.75 ± 0.01	56.7 ± 0.22	1.14 ± 0.25	11.6 ± 0.22	1.19 ± 0.19	-	23.3	74.3
**HM**	29.9 ± 2.8	0.44 ± 0.07	59.0 ± 0.06	1.16 ± 0.01	9.2 ± 0.12	0.18 ± 0.01	-	24.8	73.7
**LM**	38.3 ± 5.6	0.52 ± 0.01	49.7 ± 0.22	1.49 ± 0.04	9.6 ± 0.22	0.30 ± 0.01	-	32.5	65.5
**Commercial**	83.1 ± 2.8	0.45 ± 0.01	0.34 ± 0.10	0.08 ± 0.01	0.02 ± 0.01	1.58 ± 0.12	14.4 ± 0.2	81.4	1.5

a *dn/dC: 0.141 mL·g^–1^;**dn/dC: 0.112 mL·g^–1^. Abbreviations: GalA (galacturonic acid), Rha (rhamnose),Ara (arabinose), Xyl (xylose), Gal (galactose), Glc (glucose), Man (mannose).

b Uronic acids, determined by the Blumenkrantz and Asboe-Hansen method.^31,47^

c HG = GalA – Rha and RG-I= [GalA (%) – HG (%)] + Rha + Ara + Gal, in mol %, according to M’sakni et al.^48^

The commercial pectin displayed a high GalA content (83.1%), leading to a predominantly linear structure characterized by a high homogalacturonan (HG) content (81.4%). This structural feature is consistent with the acid-based extraction processes commonly employed for citrus pectins, which favor the formation of HG-rich chains.^46^

All samples were characterized by NMR that revealed a significant DM decrease, from ∼50% in HM to ∼27% in LM, as confirmed by the increased signal of free GalA H5 (4.71 ppm) and the concomitant decrease of MeGalA H5 (5.09 ppm). In addition, the methyl group signal (*O*Me, 3.81 ppm) showed a reduction in LM (Figure 1A).^47^ These observations are corroborated by the two-dimensional ^1^H/^13^C HSQC-NMR spectra (Figure S1).

**Figure 1:**
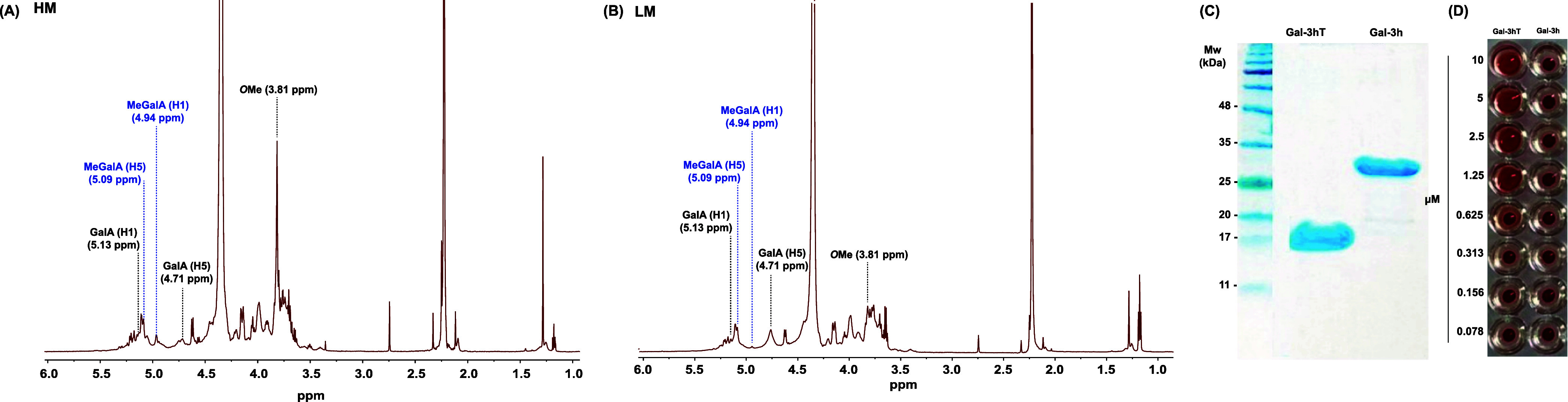
Pectin and Galectin-3 characterization. ^1^H NMR spectra of HM (A) and LM (B) pectins, SDS-PAGE electrophoretic profile of Gal-3h and Gal-3hT (C), and hemagglutination assay for Gal-3h and Gal-3hT at different concentrations (D).

Furthermore, HPSEC analysis shows that all samples display a predominant population at 37.5 min, with a molar mass range from 10^4^ to 10^6^ g mol^–1^ (Figure S2A). All samples presented the formation of colloidal systems in solution with a narrow size distribution, with low polydispersity (PDI < 0.2), with an average diameter of 30 nm. Additionally, ζ-potential analysis revealed that the net charge at the slipping plane decreased from GW to HM, LM, and the commercial sample, with values of −4.7 ± 0.1, −5.1 ± 0.2, −6.7 ± 1.1, and −17 ± 0.5 mV, respectively (Figure S2B).

The interactive potential of the pectins was evaluated in the presence of two distinct forms of Gal-3, the C-terminal domain (truncated) fragment of human galectin-3 (Gal-3hT; or CRD domain)—able to undergo crystallization for diffraction experiments—and a full-length and flexible structure (Gal-3h). The purified Gal-3hT was characterized by SDS-PAGE (Figure 1C) and displayed a molecular weight ∼17 kDa vs ∼26 kDa for the full recombinant protein (Gal-3h)—such difference is attributed to the absence of the N-terminal domain at the Gal-3hT structure, responsible for the oligomerization ability. The hemagglutination assay confirmed that only Gal-3h was able to induce mesh formation in a 2% erythrocyte solution at different concentrations (78 nM–10 μM), whereas Gal-3hT caused no aggregation effect (Figure 1D).

### 3.2 Interaction Studies

The interactive analysis between the pectins and their structural components with the proteins was initially assessed via X-ray diffraction experiments. Unfortunately, it was not possible to obtain Gal-3h crystals despite a wide range of crystallization conditions screened, possibly due to the inherent flexibility of the full-length protein.^49^ Therefore, we evaluated the interactive ability of the pectin’s constitutive monosaccharides with the Gal-3hT crystal structure.

The Gal-3hT crystals diffracted at high resolution& (from 1.1 to 1.2 Å; Figure S3 and Table S1). The crystallographic models obtained here displayed a similar topology and 3D structure compared to previous reports by presenting 11 β-sheets—divided into 5 F-face and 6 concave S-face β-sheets oppositely distributed.^50^ The native Gal-3hT crystal structure exhibited the presence of bound lactose within the binding site, loosely stabilized via hydrogen bonds performed by several residues, such as Asn160, Glu184, His158, Arg186, and Asn174 with galactose as well as the glucose moiety.^50,51^ To determine which constitutive units from the pectin structure can replace lactose from the S3–S6 residues, we soaked the Gal-3hT crystals in the following monosaccharides at 0.25 mol·L^–1^: galactose (Gal), galacturonic acid (GalA), arabinose (Ara), and rhamnose (Rha). Among them, only Gal and GalA dislocated lactose in the binding pocket as displayed in Figure 2A–C. The Gal-soaked crystal showed both Gal and lactose at the binding site, probably due to the short time used in the soaking procedure—since higher exposition times damage the crystals—but only the Gal molecule/moiety shows a clear electron density, confirming its ability to dislocate the lactose from the binding site. In particular, ligand interaction analysis revealed that both monosaccharides may perform hydrogen bonds with several residues, whereas GalA might induce the formation of salt bridges with Arg144 and Arg162. These results support that Gal and GalA units display higher affinity toward the CRD binding site and may act as key mediators along the macromolecular backbone of polysaccharides to induce Gal-3 recognition. In contrast, Ara and Rha are not related to strong associations within the CRD^52^ despite the higher amount of monosaccharides in solution.

**Figure 2:**
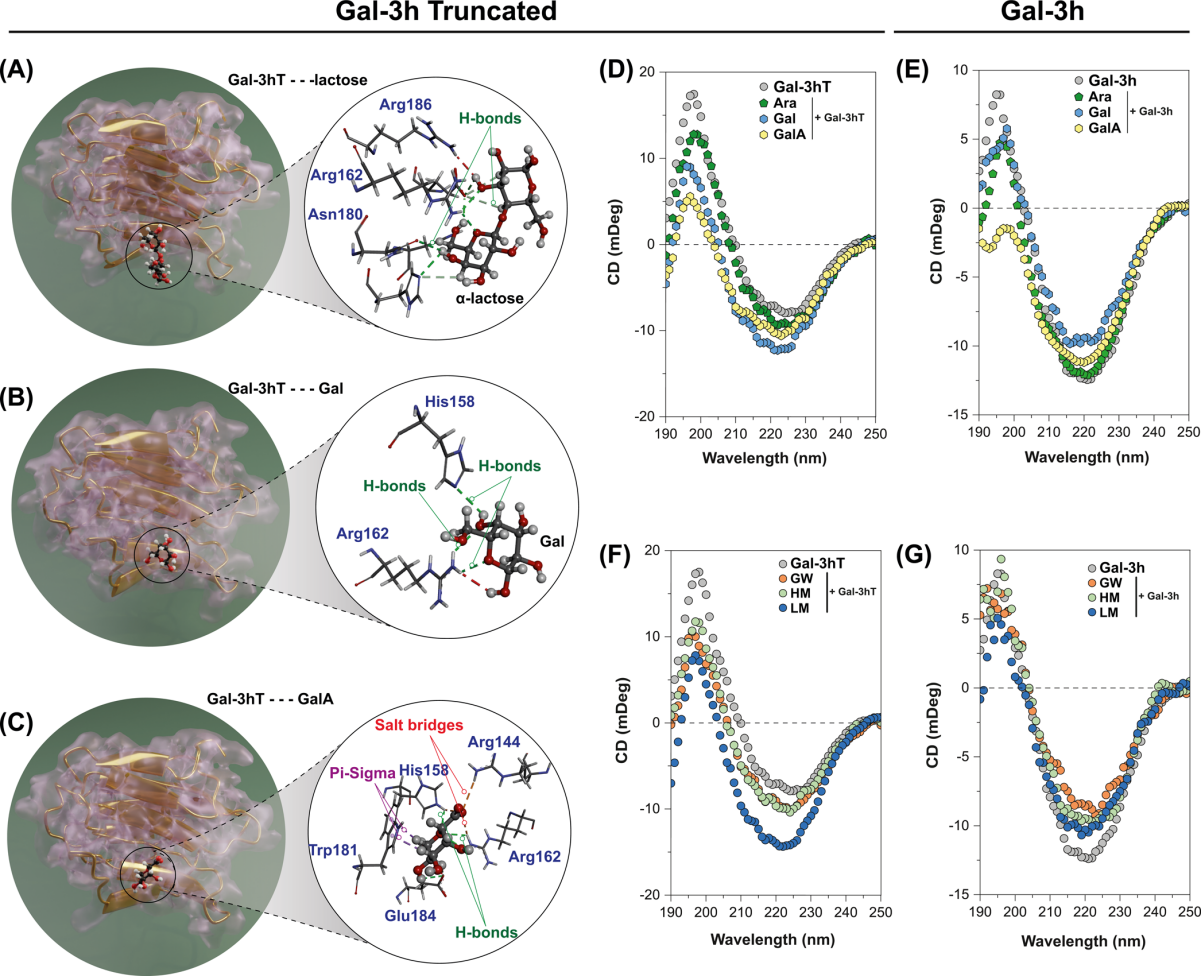
Gal-3hT and Gal-3h: pectin interactive studies. Schematic cartoon representations of X-ray diffraction analysis of the Gal-3hT crystal structure containing α-lactose (A), galactose (B), and galacturonic acid (C). The interactive region is enlarged for better visualization. Gal-3h and Gal-3hT CD spectra or in the presence of 150 μg·mL^–1^ of arabinose, galactose, or galacturonic acid (D and E). Gal-3h and Gal-3hT CD spectra alone or in the presence of 150 μg·mL^–1^ of GW, HM, or LM (F and G). Abbreviations: Ara (arabinose), Gal (galactose), GalA (galacturonic acid), Gal-3h (human galectin-3), Gal-3hT (human galectin-3 truncated). All water and solvent molecules were removed from the images to facilitate visualization.

Nonetheless, considering the size and structural nature of the large carbohydrates, the full pectins were not observed in the crystal structure after soaking. It is, however, important to highlight that the Gal-3hT solvent channels are very small (bottleneck radius: ∼3.4 Å; Matthews coefficient: ∼1.98; solvent content: ∼38%^53^), therefore, it is not expected that such large molecules could diffuse through such channels and reach the binding site. On the other hand, the binding site is located at the Gal-3hT surface, indicating that the interaction with the carbohydrate may occur in solution. Additionally, the protein in solution exhibits a higher conformational flexibility to perform ligand recognition or release and assume distinct interactive profiles.^51^

Therefore, to further analyze the interactive impact of monosaccharides as well as the pectins along the protein structure, we performed circular dichroism (CD) analysis, with Gal-3hT and Gal-3h dissolved at 0.05 mg·mL^–1^ in buffer solution at 37 °C (Figure 2D–F). The CD spectrum of both proteins is consistent with previous characterizations,^54,55^ showing a distinct secondary structure profile dominated by β-sheets. Specifically, the proteins exhibit a positive ellipticity band near 195 nm and a negative band around 215 nm, characteristic of β-sheet-rich structures. This spectral pattern suggests a topological organization involving both intra- and intersheet twists, as well as β-turns, which contribute to the overall stability and structural conformation. In fact, the presence of β-sheets is critical for maintaining the structural integrity of the protein–ligand complex, particularly in the context of lactose binding within the CRD. The natural presence of lactose in the binding pocket, following extraction and purification, further underscores the role of β-sheets in stabilizing the protein–ligand interaction.^50^

Upon the addition of monosaccharides (Ara, Gal, or GalA), the overall spectral profile of both proteins remained broadly similar to that of the lactose-bound form (Figure 2D and E), indicating that the global secondary structure of the proteins is largely preserved. Nevertheless, subtle differences were observed in the far-UV region, particularly around the characteristic β-sheet band near ∼215 nm and in the high-energy region below 200 nm. These variations suggest ligand-induced perturbations in the local organization of the β-sheet elements and neighboring secondary-structure motifs, rather than a major restructuring of the overall protein fold.

For the full-length Gal-3h protein, the presence of Gal and GalA produced noticeable spectral differences compared with the lactose-bound state.^56^ Deconvolution analysis indicated β-sheet contents of approximately 58% for the lactose-bound form, 64% in the presence of Gal, and 49% with GalA. These shifts in β-sheet content indicate that both monosaccharides induce conformational rearrangements within the protein structure. Importantly, the direction of the β-sheet variation should not be interpreted as a direct measure of binding affinity, since different ligands may promote distinct structural adaptations within the CRD while interacting with the binding site. Conversely, Ara produced only minimal spectral changes, as the CD spectral profile in the same wavelength region remained similar to that of the protein-lactose complex. This suggests a more limited interaction between Ara and the protein compared to Gal and GalA.

The effects of the polysaccharides were also evaluated for Gal-3h. In this case, the addition of GW resulted in only minor spectral changes compared with the control, whereas HM and LM induced more pronounced variations in the far-UV region (Figure 2F). The addition of GW had minimal impact on the β-sheet conformation, showing no significant shift (53.9% vs 54.3% for the protein-lactose complex). However, the presence of HM and LM pectins led to an increase in β-sheet content, reaching 64.6% and 63.2%, respectively. These findings suggest that these particular pectins can stabilize or even enhance the β-sheet structure of Gal-3h, by potentially promoting stronger interactions within the protein or between the protein and the polysaccharides.

For the truncated Gal-3hT protein, the addition of monosaccharides also resulted in detectable spectral variations (Figure 2E). The protein-lactose complex presented 58.2% of β-sheet content, and the addition of Ara, Gal, and GalA maintained the conformation with 51.8%, 52.7%, and 54.0%, respectively. The overall spectral profile remained largely consistent with that of the lactose-bound protein, indicating that the β-sheet-rich fold of the CRD domain is preserved under all conditions. The polysaccharides, on the other hand, exhibited changes in the CD signal around the ∼220 nm region for Gal-3hT, indicating a more substantial shift in secondary structure. The HM and LM pectins exhibited a greater effect, reducing the β-sheet content to 47.1% and 39.0%, respectively (Figure 2G). These spectral variations suggest that these pectins induce conformational adjustments within the truncated protein, highlighting the differential impact of polysaccharides on the full-length versus truncated forms of Gal-3h.

Overall, these results emphasize the sensitivity of the β-sheet structure in Gal-3h and Gal-3hT to various monosaccharides and α(1 → 4)-linked polysaccharides, revealing insights into the structural dynamics that govern protein-carbohydrate interactions. The correlation between X-ray diffraction and CD experiments suggests that the Gabiroba pectins with greater GalA:Ara and Gal:Ara ratios—the case of HM and LM—exhibit greater affinity toward Gal-3hT compared to GW. These data are in accordance with Zheng et al.^57^ the descriptions of GalA residues—from disaccharides and oligosaccharides—bound to Gal-3h as well as Gal-3hT and suggest an unconventional interactive orientation, where the GalA residue at the oligosaccharide reducing end flips ∼180° relative to that of the canonical β-galactoside lactose at the binding pocket.

Nonetheless, it is important to remark that, although Ara had minimal effects on protein secondary structure and was unable to displace lactose from the binding pocket, it might contribute to the anchoring process during the polysaccharide and protein interactive events. In fact, the influence on Ara content and chain length is crucial for increasing the affinity with Gal-3hT as previously reported by Wu D. et al.^52^ who verified reduced binding affinity by enzymatically removing arabinan side chains. This interpretation is consistent with the crystallographic results obtained for the CRD domain, where Gal and GalA were able to displace lactose from the carbohydrate-binding pocket, while Ara was not observed within the binding site. Moreover, HM and LM display fewer branches compared to GW based on homogalacturonan and rhamnogalacturonan content (Table 1), which may increase the exposure of the interactive backbone within the protein’s binding domains. Furthermore, the distinct CD results obtained for HM and LM might be attributed to the methyl-esterification status of GalA, indicating that the removal of *O*CH_3_ groups may elicit a more sensitive response due to an increased number of free GalA residues. This indicates that the structural features of these polysaccharides, particularly their degree of methylation and branching, play a critical role in modulating their affinity and interaction with Gal-3hT.

### 3.3 *In Vitro* Cytotoxicity and Gal-3 Expression in T98G and U87MG Cell Lineages

To further investigate the ability of the pectins to alter cell viability, we performed the MTT assay using human U87MG and T98G glioblastoma cell lines—sensitive and resistant to TMZ therapy, respectively.^58^ The LGALS3 gene overexpression was previously characterized in both cell lines by RT-qPCR.^59,60^ Accordingly, T98G and U87MG cells overexpress Gal-3 and strictly depend on the protein’s adaptive role to survive in stressful conditions, such as hypoxic and serum-deprivation media by shifting cell metabolism from oxidative phosphorylation to glycolysis in tumors.^61,62^ Moreover, recent studies highlight Gal-3 overexpression’s involvement in angiogenesis,^63^ invasion,^64^ metastasis, and poor prognosis.^65^

The MTT results revealed that all pectins induced a time and concentration-dependent cytotoxicity on both cell lines after 48 h. As displayed in Figure 3A and B, GW, HM, and LM caused a significant reduction in the percentage of metabolically active cells in concentrations above 400 μg·mL^–1^ for U87MG and above 600 μg·mL^–1^ for T98G. The commercial sample only induced cytotoxic effects at U87MG in concentrations above 500 μg·mL^–1^. In particular, the GW, HM, and LM concentrations higher than 400 μg·mL^–1^ led to >50% U87MG cell death after 48 h, whereas only HM at 1000 μg·mL^–1^ reached similar toxicity values in T98G. Therefore, the subcytotoxic dosage was defined as 300 μg·mL^–1^ for U87MG and 500 μg·mL^–1^ for T98G.

**Figure 3:**
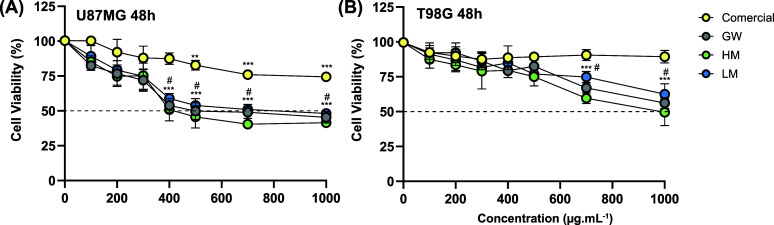
*In vitro* cytotoxicity assay. U87MG (A) and T98G (B) cell viability at 48 h as a function of Commercial, GW, HM, and LM pectins. All data are plotted as mean ± SD and statistical analyses are indexed as ***p < 0.001 and **p < 0.01 between the control and the treatment groups, and as #*p* < 0.01 between the commercial sample and GW, HM, or LM.

In a previous study,^26^ we observed that crude pectins, such as GW, significantly reduced the viability of U251MG and T98G cells, which was corroborated in this study. Interestingly, GW and other fractions maintained the growth and morphology of the embryonic mouse fibroblast cell line NIH-3T3,^26^ suggesting that the cytotoxic events may be targeted to Gal-3 overexpressing lineages. Although NIH-3T3 also expresses Gal-3,^66^ it does not display the mesenchymal and invasive character exhibited by U87MG or T98G due to the absence of distinct pathways, such as AP-1 transcriptional factors and matrix metalloproteinase-1.^67^

Despite the absence of recent studies devoted to unraveling the interactive effect between Gal-3 overexpressing GBM cell lineages and pectins, as well as their structural features, several examples explore such influence in reducing tumor cell growth and metastasis, as in the case of breast^68^ and colon cancer.^69^ The inhibitory effects caused by complex carbohydrates are likely related to dynamic and time-dependent interactions with Gal-3 and other carbohydrate-binding proteins distributed within the matrix and the cell membrane, impacting the signaling mediators associated with cell adaptation.

To further investigate these biological effects, the Gal-3 expression was determined after exposing the cells to subcytotoxic amounts of each pectin for 48 h. CLSM analysis (Figure 4A and B) displays cell populations with increased nuclear size, irregular nuclear contour, and disturbed chromatin distribution, as typically observed in tumor cells.^26^ Upon exposure to GW, HM, and LM, Gal-3 fluorescence intensity markedly decreased in both the cytoplasm and nucleus, whereas the commercial pectin caused no effect (Figure 4C and E). No evident changes in overall cell morphology were observed in T98G. However, U87MG cells exhibited a noticeable reduction in the number of adhered cells, suggesting that adhesion molecules might be dysregulated by the pectins in this cell line. This indicates that the initial response to pectin treatment may primarily involve molecular rather than structural alterations, possibly reflecting an early interference with Gal-3–dependent signaling or protein trafficking, preceding overt cytotoxicity. These results were confirmed by Western blot assays, revealing higher Gal-3 expression in the control group and a significant decrease after the treatment with GW, HM, and LM (Figure 4D,F,G and H)—with greater intensity at U87MG. At the T98G cells, only HM and LM led to a significant decrease of Gal-3 expression, which may be related to the increased resistance mechanisms displayed by this cell line.^58^

**Figure 4:**
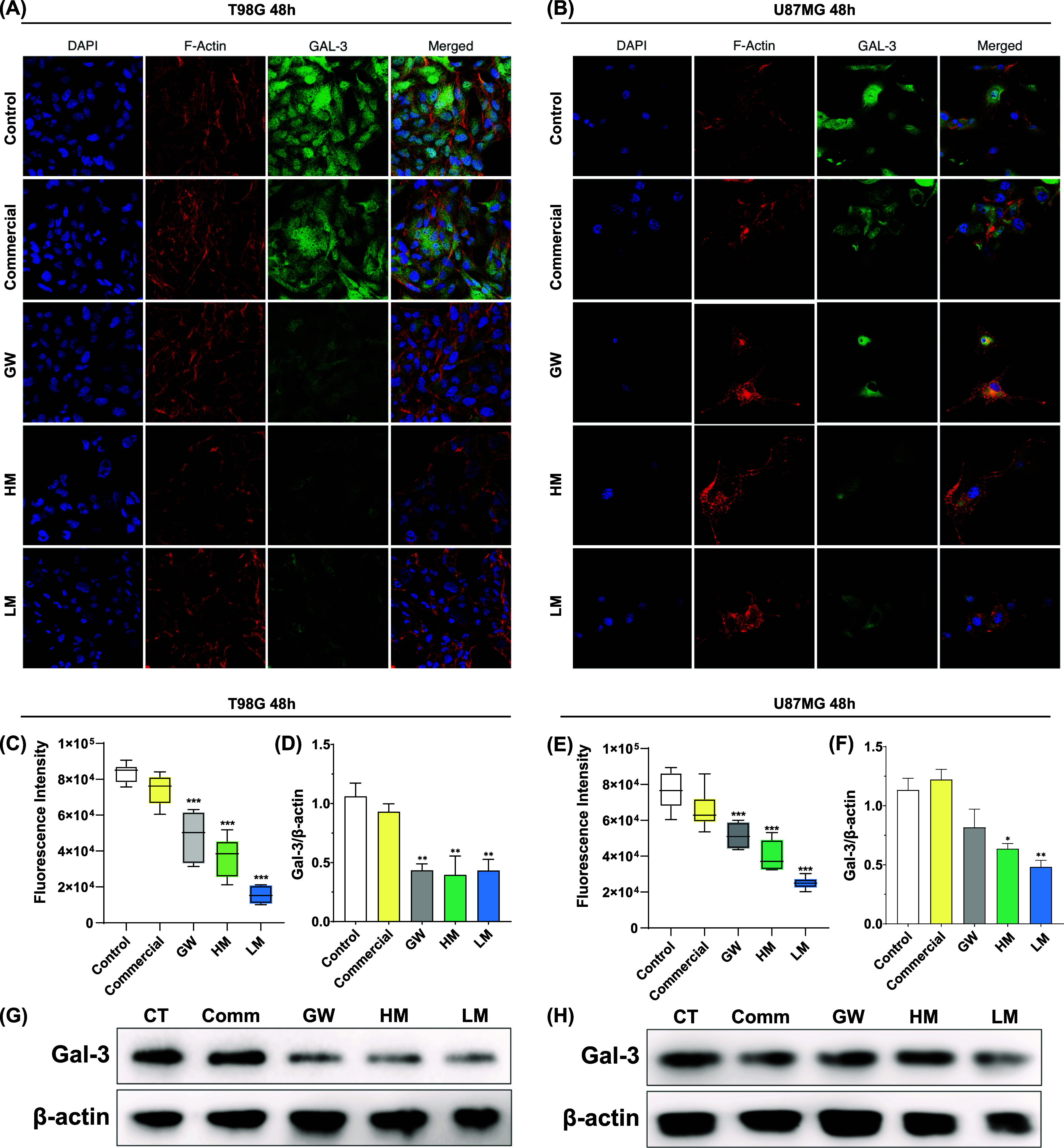
*In vitro* Gal-3 labeling and expression. Confocal laser scanning microscopy images of U87MG (A) and T98G (B) cells after 48 h of treatment with commercial, GW, HM, and LM at the subcytotoxic dose. Gal-3 fluorescence intensity for U87MG (C) and T98G (E). Western blot Gal-3/β-actin ratio and images for U87MG (D and G) and T98G (F and H), respectively. The cell nucleus is stained in blue with DAPI, the cytoskeleton in red with F-actin and Gal-3 in green. The data are plotted as mean ± SD and statistical analyses are indexed as ***p < 0.001, **p < 0.01, and *p < 0.05 compared to the control group. Additionally, U87MG cells may display variable adhesion to coverslips, particularly under certain experimental conditions, which can contribute to a lower number of cells that remain attached during fixation and staining procedures. The displayed images are representative fields from independent experiments.

The observed reduction in Gal-3 fluorescence and protein expression upon treatment with *C. xanthocarpa*-derived pectins strongly suggests that these polysaccharides interfere with Gal-3 availability or its intracellular stability in GBM cells. We hypothesize that the LM fraction, characterized by a higher GalA:Ara ratio compared to GW and HM, displays a greater accessibility to the Gal-3 CRD via its reducing-end GalA residue. Such interactions may competitively inhibit Gal-3 CRD, preventing its engagement with cell-surface glycoproteins such as integrins and laminins, thereby disrupting survival and adhesion pathways mediated by PI3K/Akt and MAPK signaling.^70^

Although the commercial sample exhibited a similarly low DM (∼27%), *M*_w_ and a comparable hydrodynamic diameter (∼30 nm, PDI > 0.2), it contained approximately 20% amidation and sucrose, with a ζ-potential of −16.7 ± 0.2 mV. Moreover, this sample showed only trace amounts of neutral monosaccharides such as Gal and Ara, being predominantly composed of GalA and displaying a highly linear structure with an HG content of 81.4% (Table 1). The lack of Gal-3 inhibition suggests that the rich Ara and Gal branching structures play a crucial role toward favoring the interaction, in accordance with Wu et al.^52^, which suggested a cooperative enrollment of RG-I and HG regions.

The differential response between U87MG and T98G cells may be attributed to their distinct molecular profiles and resistance mechanisms. U87MG cells, which are p53 wild-type^71^ and may exhibit higher Gal-3 dependency for survival, appear more susceptible to Gal-3 modulation, consistent with the pronounced downregulation observed. Conversely, T98G cells, harboring mutant p53 and enhanced NF-κB activity, may compensate for Gal-3 suppression through alternative antiapoptotic pathways. The significant Gal-3 reduction only after HM and LM treatment in T98G cells supports the notion that the de-esterified pectins exert stronger inhibitory effects due to improved molecular interaction with Gal-3 ^72^

Collectively, these data indicate that *C. xanthocarpa* pectins, particularly the HM and LM fractions, effectively downregulate Gal-3 in GMB cells through structural features that enhance their binding capacity to the Gal-3 CRD. This inhibition may contribute to the observed cytotoxicity by impairing Gal-3-mediated survival signaling and promoting a shift toward apoptotic susceptibility. Future studies integrating colocalization assays, Gal-3 silencing, and evaluation of downstream pathways will be essential to delineate the precise molecular cascade underlying these effects.

Similar results were previously reported *in vivo*, where Gal-3 inhibition led to reduced Gal-3 mRNA levels and protein expression and downregulated pathways involved in cell motility and invasion,^73^ as well as reverted the immunosuppressive state in the GBM microenvironment.^21^ Moreover, recent evidence indicates that TMZ therapy can upregulate matrix metalloproteinase-9 expression, enhancing invasiveness and contributing to poor prognosis.^74^ In this context, our findings suggest that the Gal-3-targeting pectins may serve as potential adjuvants to limit GBM progression. Given the need for local administration, future efforts will focus on developing 3D pectin-based bioimplants to assess the therapeutic impact at the GBM resection cavity.

## 4 Conclusion

In this work, pectins with distinct structures and macromolecular features obtained from *Campomanesia xanthocarpa* Berg pulp, as well as their constituent monosaccharides Gal and GalA, were investigated regarding their interactive role with the carbohydrate-binding domain of the galectin-3 protein *in vitro*. HM and LM pectins were obtained and submitted to a purification process, leading to significant changes in their monosaccharide composition, molar mass distribution, and macromolecular assembly. Structural characterization revealed that the obtained pectins are predominantly composed of Ara, GalA, and Gal, with a high proportion of RG-I regions. The interaction studies using X-ray diffraction showed that Gal and GalA have a high affinity for the Gal-3 carbohydrate recognition domain (CRD), displacing lactose from the binding site and inducing conformational changes in the protein. The circular dichroism analysis further demonstrated that HM and LM pectins significantly impact the β-sheet content of Gal-3, highlighting their potential to modulate protein structure and function. *In vitro* cytotoxicity assays revealed that HM and LM pectins induced a concentration-dependent reduction in U87MG and T98G cell viability after 48 h. The subcytotoxic dose of each pectin reduced Gal-3 expression as verified by CLSM and WB analysis. These findings suggest that the structural features of *C. xanthocarpa* pectins, such as the GalA:Ara ratio and branching, play a crucial role in their interaction with Gal-3 and their ability to inhibit GBM cell viability and reduce Gal-3 expression. Altogether, these results enable new studies based on tailored pectin structures for more potent Gal-3 inhibition or for the development of 3D-printed pectin implants after tumor-resecting surgery.

## Supplementary Material



## Notes

^1^H/^13^C HSQC NMR spectrum of HM and LM pectins. HPSEC profiles of GW, HM, LM, and commercial pectins. Number-weight hydrodynamic diameter and Zeta-potential analysis of all pectins. Microscopic image of gal-3hT crystals prepared for X-ray diffraction experiments. X-ray diffraction data collection and refinement statistics. (PDF)

## References

[1] Eisenbarth D., Wang Y. A. (2023). Glioblastoma heterogeneity at single cell resolution. Oncogene.

[2] Lah T. T., Novak M., Breznik B. (2020). Brain malignancies:
Glioblastoma
and brain metastases. Semin. Cancer Biol..

[3] Ferlay J., Colombet M., Soerjomataram I., Mathers C., Parkin D. M., Piñeros M., Znaor A., Bray F. (2019). Estimating the global
cancer incidence and mortality in 2018: GLOBOCAN sources and methods. Int. J. Cancer.

[4] Louis D. N., Perry A., Reifenberger G., Von Deimling A., Figarella-Branger D., Cavenee W. K., Ohgaki H., Wiestler O. D., Kleihues P., Ellison D. W. (2016). The 2016 World Health
Organization
classification of tumors of the central nervous system: A summary. Acta Neuropathol..

[5] Omuro A., DeAngelis L. M. (2013). Glioblastoma and Other Malignant
Gliomas: A Clinical
Review. JAMA.

[6] Klemm F., Möckl A., Salamero-Boix A., Alekseeva T., Schäffer A., Schulz M., Niesel K., Maas R. R., Groth M., Elie B. T. (2021). Compensatory CSF2-driven
macrophage activation promotes adaptive resistance to CSF1R inhibition
in breast-to-brain metastasis. Nat. Cancer.

[7] Quail D. F., Joyce J. A. (2017). The microenvironmental
landscape of brain tumors. Cancer Cell.

[8] Tondepu C., Karumbaiah L. (2022). Glycomaterials to investigate the functional role of
aberrant glycosylation in Glioblastoma. Adv.
Healthcare Mater..

[9] Girotti M. R., Salatino M., Dalotto-Moreno T., Rabinovich G. A. (2020). Sweetening
the hallmarks of cancer: Galectins as multifunctional mediators of
tumor progression. J. Exp. Med..

[10] Pace A., Scirocchi F., Napoletano C., Zizzari I. G., D’Angelo L., Santoro A., Nuti M., Rahimi H., Rughetti A. (2022). Glycan-Lectin
Interactions as Novel Immunosuppression Drivers in Glioblastoma. Int. J. Mol. Sci..

[11] Raposo C. D., Canelas A. B., Barros M. T. (2021). Human lectins, their carbohydrate
affinities and where to find them. Biomolecules.

[12] Thiemann S., Baum L. G. (2016). Galectins and immune
responses—just how do they
do those things they do?. Annu. Rev. Immunol..

[13] Wang H., Song X., Huang Q., Xu T., Yun D., Wang Y., Hu L., Yan Y., Chen H., Lu D. (2019). LGALS3 promotes treatment resistance in glioblastoma
and is associated with tumor risk and prognosis. Cancer Epidemiol. Biomarkers Prev..

[14] Dumic J., Dabelic S., Flögel M. (2006). Galectin-3:
An open-ended story. Biochim. Biophys. Acta
- Gen. Subj..

[15] Ahmed R., Anam K., Ahmed H. (2023). Development of Galectin-3
Targeting
Drugs for Therapeutic Applications in Various Diseases. Int. J. Mol. Sci..

[16] Mariño K. V., Cagnoni A. J., Croci D. O., Rabinovich G. A. (2023). Targeting
galectin-driven regulatory circuits in cancer and fibrosis. Nat. Rev. Drug Discovery.

[17] Ahmad N., Gabius H.-J., André S., Kaltner H., Sabesan S., Roy R., Liu B., Macaluso F., Brewer C. F. (2004). Galectin-3 precipitates
as a pentamer with synthetic multivalent carbohydrates and forms heterogeneous
cross-linked complexes. J. Biol. Chem..

[18] Farhadi S. A., Liu R., Becker M. W., Phelps E. A., Hudalla G. A. (2021). Physical tuning
of galectin-3 signaling. Proc. Natl. Acad. Sci.
U. S. A..

[19] Zhao Z., Xu X., Cheng H., Miller M. C., He Z., Gu H., Zhang Z., Raz A., Mayo K. H., Tai G. (2021). Galectin-3 N-terminal tail prolines modulate cell activity and glycan-mediated
oligomerization/phase separation. Proc. Natl.
Acad. Sci. U. S. A..

[20] Seguin L., Odouard S., Corlazzoli F., Haddad S. A., Moindrot L., Calvo Tardón M., Yebra M., Koval A., Marinari E., Bes V. (2021). Macropinocytosis requires Gal-3 in a subset of patient-derived
glioblastoma stem cells. Commun. Biol..

[21] Rivera-Ramos A., Cruz-Hernández L., Talaverón R., Sánchez-Montero M. T., García-Revilla J., Mulero-Acevedo M., Deierborg T., Venero J. L., Sarmiento
Soto M. (2024). Galectin-3 depletion tames pro-tumoural microglia and restrains cancer
cells growth. Cancer Lett..

[22] Zhang T., Miller M. C., Zheng Y., Zhang Z., Xue H., Zhao D., Su J., Mayo K. H., Zhou Y., Tai G. (2017). Macromolecular assemblies of complex polysaccharides with galectin-3
and their synergistic effects on function. Biochem.
J..

[23] Roman-Benn A., Contador C. A., Li M.-W., Lam H.-M., Ah-Hen K., Ulloa P. E., Ravanal M. C. (2023). Pectin: An overview of sources, extraction
and applications in food products, biomedical, pharmaceutical and
environmental issues. Food Chem. Adv..

[24] Voragen A. G., Coenen G.-J., Verhoef R. P., Schols H. A. (2009). Pectin, a versatile
polysaccharide present in plant cell walls. Struct. Chem..

[25] Yapo B. M. (2011). Pectic
substances: From simple pectic polysaccharides to complex pectins—A
new hypothetical model. Carbohydr. Polym..

[26] da
Costa Amaral S., Barbieri S. F., Ruthes A. C., Bark J. M., Winnischofer S. M. B., Silveira J. L. M. (2019). Cytotoxic effect of crude and purified
pectins from Campomanesia xanthocarpa Berg on human glioblastoma cells. Carbohydr. Polym..

[27] Barbieri S. F., Ruthes A. C., Petkowicz C. L. D. O., de Godoy R. C. B., Sassaki G. L., Santana
Filho A. P., Silveira J. L. M. (2017). Extraction, purification and structural
characterization of a galactoglucomannan from the gabiroba fruit (Campomanesia
xanthocarpa Berg), Myrtaceae family. Carbohydr.
Polym..

[28] Barbieri S. F., da Costa Amaral S., Mazepa E., Filho A. P. S., Sassaki G. L., Silveira J. L. M. (2022). Isolation, NMR characterization and bioactivity of
a (4-O-methyl-α-D-glucurono)-β-D-xylan from Campomanesia
xanthocarpa Berg fruits. Int. J. Biol. Macromol..

[29] da
Costa Amaral S., Roux D., Caton F., Rinaudo M., Barbieri S. F., Meira Silveira J. L. (2021). Extraction, characterization and
gelling ability of pectins from Araçá (Psidium cattleianum
Sabine) fruits. Food Hydrocolloids.

[30] Wolfrom M., Thompson A., Lineback D. (1963). Isopropyl
Tetra-O-acetyl-α-D-glucopyranoside;
A Synthesis of Kojibiose. J. Org. Chem..

[31] Blumenkrantz N., Asboe-Hansen G. (1973). New method for quantitative determination of uronic
acids. Anal. Biochem..

[32] Grasdalen H., Einar Bakøy O., Larsen B. (1988). Determination of the degree of esterification
and the distribution of methylated and free carboxyl groups in pectins
by 1H-n.m.r. spectroscopy. Carbohydr. Res..

[33] Biscaia S. M. P., Pires C., Lívero F. A. R., Bellan D. L., Bini I., Bustos S. O., Vasconcelos R. O., Acco A., Iacomini M., Carbonero E. R. (2022). MG-Pe: A Novel Galectin-3 Ligand with Antimelanoma
Properties and Adjuvant Effects to Dacarbazine. Int. J. Mol. Sci..

[34] Laemmli U. K. (1970). Cleavage
of structural proteins during the assembly of the head of bacteriophage
T4. Nature.

[35] Bradford M. M. (1976). A rapid
and sensitive method for the quantitation of microgram quantities
of protein utilizing the principle of protein-dye binding. Anal. Biochem..

[36] Si Y., Li Y., Yang T., Li X., Ayala G. J., Mayo K. H., Tai G., Su J., Zhou Y. (2021). Structure–function studies
of galectin-14, an important effector molecule in embryology. FEBS J..

[37] Kabsch W. (2010). xds. Acta Crystallogr.,
Sect. D: Biol. Crystallogr..

[38] Karplus P. A., Diederichs K. (2012). Linking crystallographic
model and data quality. Science.

[39] McCoy A. J., Grosse-Kunstleve R. W., Adams P. D., Winn M. D., Storoni L. C., Read R. J. (2007). Phaser crystallographic software. J. Appl. Crystallogr..

[40] Emsley P., Lohkamp B., Scott W. G., Cowtan K. (2010). Features and development
of Coot. Acta Crystallogr., Sect. D: Biol. Crystallogr..

[41] Afonine P. V., Grosse-Kunstleve R. W., Echols N., Headd J. J., Moriarty N. W., Mustyakimov M., Terwilliger T. C., Urzhumtsev A., Zwart P. H., Adams P. D. (2012). Towards automated crystallographic
structure refinement with phenix. refine. Acta
Crystallogr., Sect. D: Biol. Crystallogr..

[42] Williams C. J., Headd J. J., Moriarty N. W., Prisant M. G., Videau L. L., Deis L. N., Verma V., Keedy D. A., Hintze B. J., Chen V. B. (2018). MolProbity:
More and better reference data for improved
all-atom structure validation. Protein Sci..

[43] Micsonai A., Moussong É., Wien F., Boros E., Vadászi H., Murvai N., Lee Y.-H., Molnár T., Réfrégiers M., Goto Y. (2022). BeStSel:
Webserver for secondary structure and fold prediction for protein
CD spectroscopy. Nucleic Acids Res..

[44] Barbieri S. F., da Costa Amaral S., Ruthes A. C., de Oliveira Petkowicz C. L., Kerkhoven N. C., da Silva E. R. A., Silveira J. L. M. (2019). Pectins from
the pulp of gabiroba (Campomanesia xanthocarpa Berg): Structural characterization
and rheological behavior. Carbohydr. Polym..

[45] Ponder G., Richards G. (1997). Arabinogalactan from
Western larch, Part III: Alkaline
degradation revisited, with novel conclusions on molecular structure. Carbohydr. Polym..

[46] Li H., Li Z., Wang P., Liu Z., An L., Zhang X., Xie Z., Wang Y., Li X., Gao W. (2024). Evaluation of citrus
pectin extraction methods: Synergistic enhancement of pectin’s
antioxidant capacity and gel properties through combined use of organic
acids, ultrasonication, and microwaves. Int.
J. Biol. Macromol..

[47] Dias I. P., Barbieri S. F., da Costa Amaral S., Silveira J. L. M. (2024). Development and
characterization of films from Campomanesia xanthocarpa and commercial
citrus pectins with different degrees of methyl-esterification. Int. J. Biol. Macromol..

[48] M’sakni N. H., Majdoub H., Roudesli S., Picton L., Le Cerf D., Rihouey C., Morvan C. (2006). Composition, structure and solution
properties of polysaccharides extracted from leaves of Mesembryanthenum
crystallinum. Eur. Polym. J..

[49] Flores-Ibarra A., Vértesy S., Medrano F. J., Gabius H.-J., Romero A. (2018). Crystallization
of a human galectin-3 variant with two ordered segments in the shortened
N-terminal tail. Sci. Rep..

[50] Seetharaman J., Kanigsberg A., Slaaby R., Leffler H., Barondes S. H., Rini J. M. (1998). X-ray crystal structure of the human
galectin-3 carbohydrate
recognition domain at 2.1-Å resolution. J. Biol. Chem..

[51] Saraboji K., Håkansson M., Genheden S., Diehl C., Qvist J., Weininger U., Nilsson U. J., Leffler H., Ryde U., Akke M., Logan D. T. (2012). The Carbohydrate-Binding Site in
Galectin-3 Is Preorganized To Recognize a Sugarlike Framework of Oxygens:
Ultra-High-Resolution Structures and Water Dynamics. Biochemistry.

[52] Wu D., Zheng J., Hu W., Zheng X., He Q., Linhardt R. J., Ye X., Chen S. (2020). Structure-activity
relationship of Citrus segment membrane RG-I pectin against Galectin-3:
The galactan is not the only important factor. Carbohydr. Polym..

[53] Pletzer-Zelgert J., Ehrt C., Fender I., Griewel A., Flachsenberg F., Klebe G., Rarey M. (2023). LifeSoaks: A tool for analyzing solvent
channels in protein crystals and obstacles for soaking experiments. Acta Crystallogr. D Struct Biol..

[54] Pirone L., Lenza M. P., Di Gaetano S., Capasso D., Filocaso M., Russo R., Di Carluccio C., Saviano M., Silipo A., Pedone E. (2024). Biophysical and Structural
Characterization of the
Interaction between Human Galectin-3 and the Lipopolysaccharide from
Pseudomonas aeruginosa. Int. J. Mol. Sci..

[55] Duckworth C. A., Guimond S. E., Sindrewicz P., Hughes A. J., French N. S., Lian L.-Y., Yates E. A., Pritchard D. M., Rhodes J. M., Turnbull J. E. (2015). Chemically
modified,
non-anticoagulant heparin derivatives are potent galectin-3 binding
inhibitors and inhibit circulating galectin-3-promoted metastasis. Oncotarget.

[56] Kelly S. M., Jess T. J., Price N. C. (2005). How to study proteins
by circular
dichroism. Biochim. Biophys. Acta, Proteins
Proteomics.

[57] Zheng Y., Su J., Miller M. C., Geng J., Xu X., Zhang T., Mayzel M., Zhou Y., Mayo K. H., Tai G. (2021). Topsy-turvy
binding of negatively charged homogalacturonan oligosaccharides to
galectin-3. Glycobiology.

[58] Kochanowski P., Catapano J., Pudełek M., Wróbel T., Madeja Z., Ryszawy D., Czyż J. (2021). Temozolomide
induces the acquisition of invasive phenotype by o6-methylguanine-dna
methyltransferase (Mgmt)+ glioblastoma cells in a snail-1/cx43-dependent
manner. Int. J. Mol. Sci..

[59] Lahm H., André S., Hoeflich A., Fischer J. R., Sordat B., Kaltner H., Wolf E., Gabius H.-J. (2001). Comprehensive galectin
fingerprinting in a panel of 61 human tumor cell lines by RT-PCR and
its implications for diagnostic and therapeutic procedures. J. Cancer Res. Clin. Oncol..

[60] McClung H. M., Thomas S. L., Osenkowski P., Toth M., Menon P., Raz A., Fridman R., Rempel S. A. (2007). SPARC upregulates MT1-MMP expression,
MMP-2 activation, and the secretion and cleavage of galectin-3 in
U87MG glioma cells. Neurosci. Lett..

[61] Ikemori R. Y., Machado C. M. L., Furuzawa K. M., Nonogaki S., Osinaga E., Umezawa K., de Carvalho M. A., Verinaud L., Chammas R. (2014). Galectin-3
up-regulation in hypoxic and nutrient deprived microenvironments promotes
cell survival. PLoS One.

[62] Li Y.-S., Li X.-T., Yu L.-G., Wang L., Shi Z.-Y., Guo X.-L. (2020). Roles of galectin-3
in metabolic disorders and tumor
cell metabolism. Int. J. Biol. Macromol..

[63] Dos
Santos S. N., Sheldon H., Pereira J. X., Paluch C., Bridges E. M., El-Cheikh M. C., Harris A. L., Bernardes E. S. (2017). Galectin-3
acts as an angiogenic switch to induce tumor angiogenesis via Jagged-1/Notch
activation. Oncotarget.

[64] Li S., Pritchard D. M., Yu L.-G. (2023). Galectin-3 promotes secretion of
proteases that decrease epithelium integrity in human colon cancer
cells. Cell Death Dis..

[65] Song M., Pan Q., Yang J., He J., Zeng J., Cheng S., Huang Y., Zhou Z.-Q., Zhu Q., Yang C., Han Y., Tang Y., Chen H., Weng D.-S., Xia J.-C. (2020). Galectin-3
favours tumour metastasis via the activation of β-catenin signalling
in hepatocellular carcinoma. Br. J. Cancer.

[66] Takenaka Y., Fukumori T., Yoshii T., Oka N., Inohara H., Kim H.-R. C., Bresalier R. S., Raz A. (2004). Nuclear export of phosphorylated
galectin-3 regulates its antiapoptotic activity in response to chemotherapeutic
drugs. Mol. Cell. Biol..

[67] Wang Y.-G., Kim S.-J., Baek J.-H., Lee H.-W., Jeong S.-Y., Chun K.-H. (2012). Galectin-3 increases
the motility of mouse melanoma
cells by regulating matrix metalloproteinase-1 expression. Exp. Mol. Med..

[68] Wang L., Li Y.-S., Yu L.-G., Zhang X.-K., Zhao L., Gong F.-L., Yang X.-X., Guo X.-L. (2020). Galectin-3 expression
and secretion by tumor-associated macrophages in hypoxia promotes
breast cancer progression. Biochem. Pharmacol..

[69] Do
Nascimento R. S., Pedrosa L. D. F., Diethelm L. T. H., Souza T., Shiga T. M., Fabi J. P. (2020). The purification of pectin from commercial
fruit flours results in a jaboticaba fraction that inhibits galectin-3
and colon cancer cell growth. Food Res. Int..

[70] Gao X., Balan V., Tai G., Raz A. (2014). Galectin-3 induces
cell migration via a calcium-sensitive MAPK/ERK1/2 pathway. Oncotarget.

[71] Villalonga-Planells R., Coll-Mulet L., Martinez-Soler F., Castano E., Acebes J.-J., Gimenez-Bonafe P., Gil J., Tortosa A. (2011). Activation of p53 by
nutlin-3a induces apoptosis and cellular senescence in human glioblastoma
multiforme. PLoS One.

[72] Zhang T., Sun G., Shuai M., Ye J., Huang J., Yao X., Sun C., Min X. (2021). Purification,
chemical analysis and inhibitory effects
on galectin-3 of enzymatic pH-modified citrus pectin. Food Chem..

[73] Pan X., Wang H., Zheng Z., Huang X., Yang L., Liu J., Wang K., Zhang Y. (2022). Pectic polysaccharide from Smilax
china L. ameliorated ulcerative colitis by inhibiting the galectin-3/NLRP3
inflammasome pathway. Carbohydr. Polym..

[74] Thanh H. D., Lee S., Nguyen T. T., Huu T. N., Ahn E.-J., Cho S.-H., Kim M. S., Moon K.-S., Jung C. (2024). Temozolomide promotes
matrix metalloproteinase 9 expression through p38 MAPK and JNK pathways
in glioblastoma cells. Sci. Rep..

