# Supplementary material for: Exploring the Interactive Effect of Pectins from Campomanesia xanthocarpa with Galectin‑3
Source: ACS Omega. 2026 Apr 2;11(14):22379–90. doi: 10.1021/acsomega.6c01637 (PMC13084385; doi:10.1021/acsomega.6c01637)
Supplement: Supplementary file 1 [file ao6c01637_si_001.pdf]

Supplementary Information

Exploring the interactive effect of pectins from *Campomanesia xanthocarpa* with galectin-3

Isabela P. Dias[1,T], Gabriela C. Cardoso[5,T], Lucas V. dos Santos[1], Giovanna Furman[1], Ester Mazepa[1], Téo F. Minella[2], Marcelo D. Baruffi[3], Luisa Mestriner[3], Andrey F. Z. Nascimento[4], Lindomar J. C. Albuquerque[4], Keylla L. Mischiatti[2], Edneia A. S. Ramos[5,6], Elaine C. de A. Abreu[7], Sheila M. B. Winnischofer[1,2], Sarah da Costa Amaral[1], Joana Léa Meira Silveira[1,*], Guilherme F. Picheth[1,2,*]

[1]Postgraduate Program in Biochemistry Sciences, Federal University of Paraná, Curitiba, PR, Brazil.
[2]Department of Biochemistry and Molecular Biology, Federal University of Paraná, Curitiba, PR, Brazil.
[3]Department of Clinical, Toxicological and Bromatological Analysis, University of São Paulo, Ribeirão Preto, SP, Brazil.
[4]Brazilian Synchrotron Light Laboratory, Brazilian Center for Research in Energy and Materials, Campinas, SP, Brazil.
[5]Post-graduate Program in Microbiology, Parasitology and Pathology, Federal University of Paraná, Curitiba, PR, Brazil
[6]Department of Basic Pathology, Federal University of Paraná, Curitiba, PR, Brazil.
[7] Center for Advanced Fluorescence Technologies from the Federal University of Paraná - CTAF/UFPR, Curitiba, PR, Brazil.

[T] These authors contributed equally.

*Corresponding authors

Joana Léa Meira Silveira. Postgraduate Program in Biochemistry Sciences, Sector of Biological Sciences, Federal University of Paraná, Curitiba, PR, Brazil.

Email address: jlms12@yahoo.com

Guilherme F. Picheth. Department of Biochemistry, Federal University of Paraná. Curitiba, PR, Brazil.

Email address: gfpicheth@ufpr.br

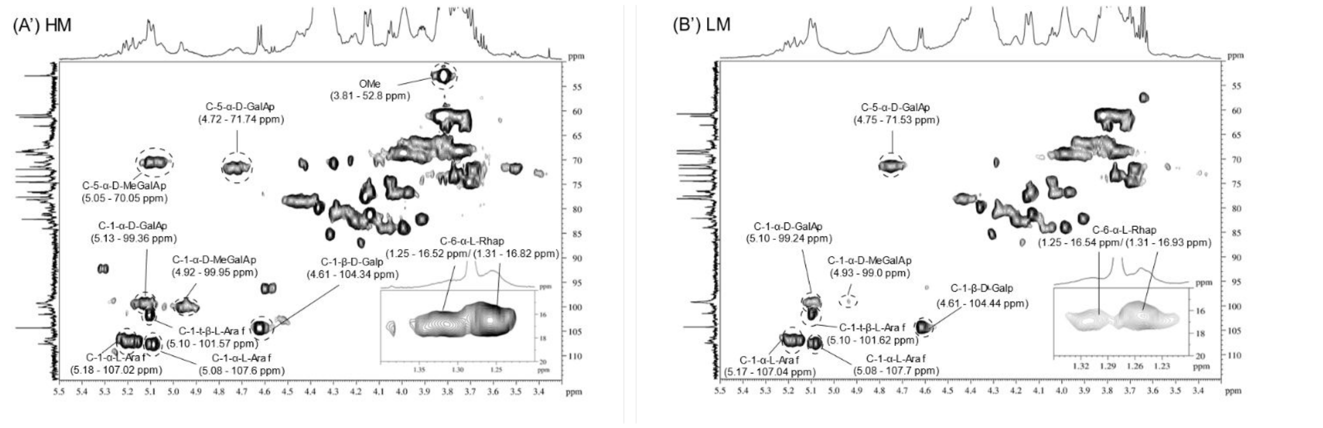

Figure S1. $^{1}H/^{13}C$ HSQC - NMR spectrum of the (A) HM pectin and (B) LM pectin samples. The samples were dissolved in D_2O and data were collected at a probe temperature of 70 °C.

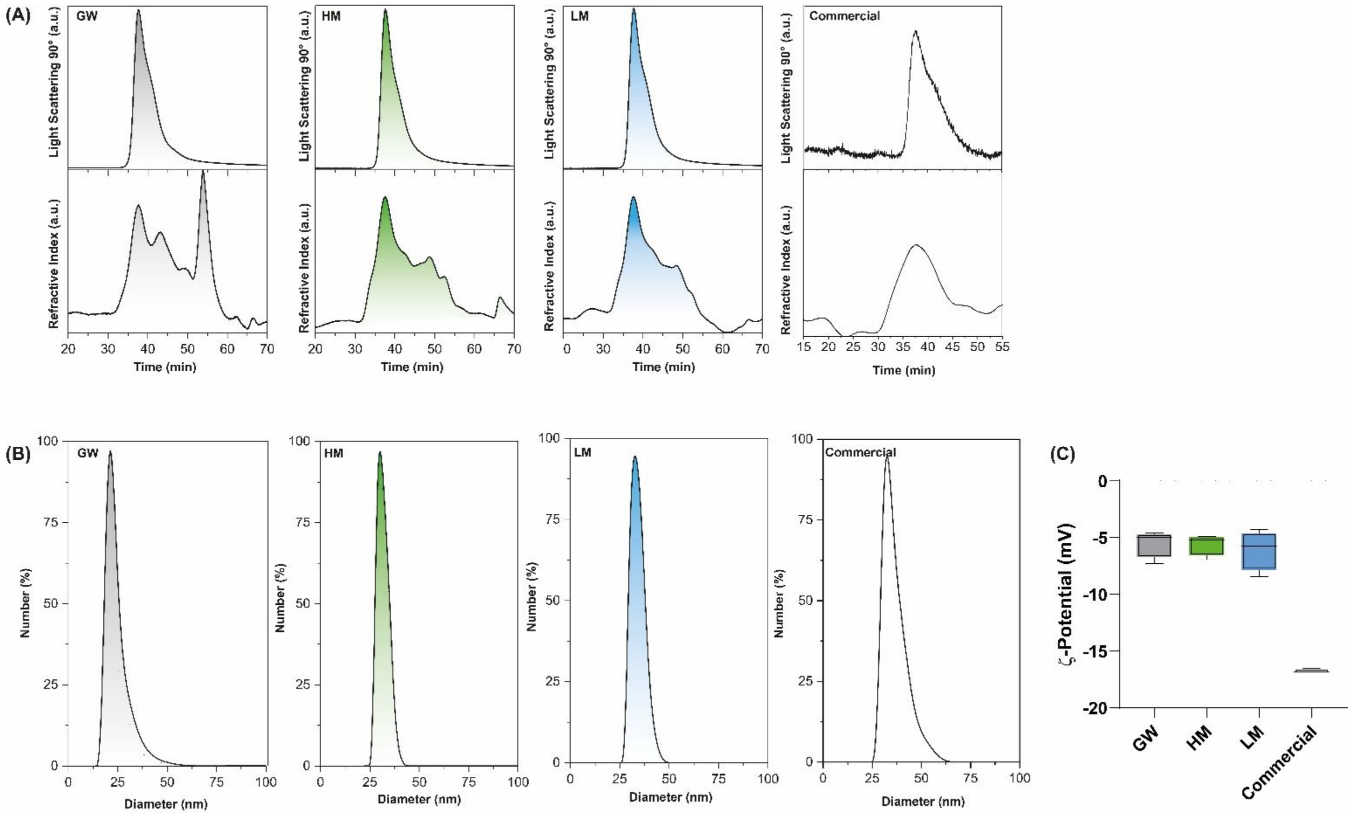

Figure S2. 90° light scattering and refractive index intensity for HPSEC profiles of GW, HM, LM and commercial pectins (A). Number-weight hydrodynamic diameter (B) and Zeta-potential analysis of all pectins (B).

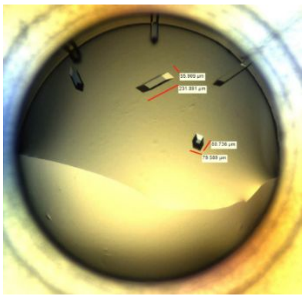

Figure S3. Representative microscopic image of gal-3hT crystals prepared for X-ray diffraction experiments.

Table S1. Data collection and refinement statistics.

	gal-3hT Lac (native)	gal-3hT Gal soak	gal-3hT GalA soak
**Wavelength (Å)**	0.97718	0.97718	0.97718
**Resolution range (Å)**	47.76-1.1 (1.139-1.1)	42.46-1.15 (1.191-1.15)	41.45-1.2 (1.243-1.2)
**Space group**	$P\ 2_1\ 2_1\ 2_1$	$P\ 2_1\ 2_1\ 2_1$	$P\ 2_1\ 2_1\ 2_1$
**Unit cell (Å; °)**	34.517 57.016 61.342 α=β=γ=90°	35.886 57.798 62.573 α=β=γ=90°	34.293 56.261 61.298 α=β=γ=90°
**Total reflections**	567497 (20675)	574488 (43523)	475633 (45555)
**Unique reflections**	49254 (4274)	45780 (4117)	37836 (3714)
**Multiplicity**	11.5 (4.8)	12.5 (10.6)	12.6 (12.3)
**Completeness (%)**	98.66 (87.44)	97.37 (89.13)	99.97 (100.00)
**Mean I/sigma(I)**	24.92 (2.93)	22.90 (3.81)	13.95 (1.08)
**Wilson B-factor**	10.58	11.29	15.54
**R-meas**	0.04953 (0.4579)	0.06118 (0.6362)	0.09123 (2.513)
**CC1/2**	0.999 (0.873)	0.999 (0.929)	0.999 (0.513)
**Refl. used in refinement**	49243 (4274)	45780 (4115)	37836 (3714)
**Refl. used for R-free**	2463 (214)	2289 (205)	1892 (185)
**R-work**	0.1298 (0.2009)	0.1180 (0.1411)	0.1396 (0.2582)
**R-free**	0.1510 (0.2410)	0.1365 (0.1572)	0.1642 (0.2925)
**Num. of non-H atoms**	1452	1395	1335
**macromolecules**	1181	1159	1145
**ligands**	142	69	23
**solvent**	202	200	176
**Protein residues**	139	139	139
**RMS(bonds)**	0.010	0.013	0.005
**RMS(angles)**	1.11	1.17	0.92
**Ramachandran**			
**favored (%)**	98.54	98.54	98.54
**allowed (%)**	1.46	1.46	1.46
**outliers (%)**	0.00	0.00	0.00
**Rotamer outliers (%)**	1.46	0.75	0.00
**Clashscore**	8.96	4.52	3.83
**Average B-factor**	16.83	16.53	22.91
**macromolecules**	13.58	14.04	20.55
**ligands**	25.83	21.21	28.79
**solvent**	32.79	30.15	37.83

Statistics for the highest-resolution shell are shown in parentheses. Refl, reflections.
